# Shared Decision Making, cognition, participation and heath of women during acute psychiatric hospitalization

**DOI:** 10.1192/j.eurpsy.2026.10769

**Published:** 2026-07-31

**Authors:** E. Lipskaya-Velikovsky, S. Ahrend, A. Juven-Wetzler, Y. Zisman-Ilani

**Affiliations:** 1 The School of Occupational Therapy, The Hebrew University; 2 Occupational Therapy, Jerusalem Mental Health Center; 3 The Faculty of Medicine, The Hebrew University, Jerusalem, Israel; 4 College of Public Health, Temple University, Philadelphia, United States

## Abstract

**Introduction:**

Shared Decision Making (SDM) focuses on patient–clinician interactions regarding treatment decisions, aiming to improve health outcomes through personally tailored care. SDM aligns with recovery-oriented care, emphasizing self-determination, choice, and autonomy and therefore is increasingly used in community-based mental health services worldwide, supported by ethical, clinical, and empirical evidence. Yet, SDM is not systematically implemented and investigated during psychiatric hospitalization. Barriers to inpatient SDM include concerns about inpatients’ limited decision-making capacity and cognitive impairments, which may be especially prominent for women due to gender and cultural factors.

**Objectives:**

This study aimed to examine, for the first time, association between SDM and cognition, participation in daily life, and health indices among women hospitalized in an inpatient psychiatric setting.

**Methods:**

This cross-sectional study included 38 women (Mdn = 33 years, IQR = 25–42) hospitalized in a psychiatric acute facility. Psychotic disorders were the most common diagnosis (N = 28, 73.7%), with a median illness onset at 22.5 years (IQR = 18–26.5). Most participants were voluntarily hospitalized (N = 24, 63.2%) and were candidates for discharge at the time of data collection (N = 23, 60.5%). All the participants completed the following assessments: Shared Decision Making Questionnaire-9-Psy (SDM-Q-9-Psy) to evaluate experience of involvement in the decision-making process; Montreal Cognitive Assessment for cognitive evaluation; the Kohlman Evaluation of Living Skills (KELS) for functional capacity; the Engagement in Meaningful Activities Survey (EMAS) for meaningful participation; and the Patient Health Questionnaire-9 (PHQ-9) for self-evaluation of health. In addition, the Brief Psychiatric Rating Scale (BPRS) was completed by a clinician for each participant to evaluate the severity of psychiatric symptoms.

**Results:**

SDM-Q-9-Psy scores were relatively low (Mdn = 28.5, IQR = 16–41.5). At average, the participants experienced moderate psychiatric symptoms (BPRS: M=46.5, SD 14) and slight cognitive impairments (MoCA: M=23.4; SD=3.8), however, functionally could be independent (KELS: Mdn=2.5, IQR: 1-4.5). Lower SDM-Q-9-Psy score was associated with longer hospitalization (r = –0.367, p < .05) and higher BPRS scores (r =–.334, p<.05). No significant correlations were found between SDM-Q-9-Psy and cognitive functioning, functional capacity, participation, or self-rated health (–0.3<r<0.16, p>.05).

**Conclusions:**

Among women in acute psychiatric wards, SDM was compromised and associated with illness-related variables―symptom severity and hospitalization length― regardless of personal capacities or self-rated health. These findings raise concern about personally tailored practice and SDM implementation in acute psychiatric care.

**Disclosure of Interest:**

None Declared

